# Hospital and Institutional News

**Published:** 1914-05-09

**Authors:** 


### HOSPITAL AND INSTITUTIONAL NEWS.

### THE SUPERINTENDENTSHIP AT ST. GEORGE'S.

Dr. J. Nachbar has been elected to the post of medical superintendent at St. George's Hospital. It is understood that there were about twenty-six candidates for this post, several of whom had but slender chances of securing support. According to current report, which we give solely for what it is worth, one of the candidates was a medical man who is also in holy orders, and is distinguished as a yachtsman and a black-and-white artist; another a London County Council medical officer, lately physician at a London children's hospital; and another the gentleman who has been carrying out the duties temporarily since Mr. Friend's resignation last summer. Mr. Nachbar has a great opportunity of hard work to rise to, and we hope that he will take it to the full. He is an official of the Royal Society of Medicine who was resident medical officer at St. George's some years ago.

### THE NEW BUDGET AND THE HOSPITALS.

One feature of Mr. Lloyd George's new Budget is the help for local authorities, which, under specified conditions, he has brought forward in the attempt to relieve, on the one hand, the present incidence of the rates, and to prevent many Acts of Parliament affecting local concerns remaining, from their prospective costs, and in view of already high rates, a dead letter. The new grants to localities include the optional provision of £45,000 under the heading of Mental Deficiency; £1,300,000 for the first year, rising to £4,000,000, under the heading of Public Health; and £750,000 for Tuberculosis, Nursing, and Pathological Laboratories. Here we get, at all events, an instalment of Government aid for research, but between the promise of a grant and its healthy effect on institutional activity are many pitfalls, among which we need mention here merely the method of allocation, and the complicated points involved where research is so much decentralised as it is in this country at present. The Government, it seems, are also to contribute one moiety to the cost of feeding school children, and grants are to be made for maternity centres—in which connection we may refer our readers to the striking article which appeared in The Hospital only last week (page 132) on "The State and the Environment of Pregnancy"—for open-air schools, and for physical training. It is easy to draw the general moral from these somewhat belated attempts to follow the direction which medical men have been pointing out insistently for years—namely, that if the profession by employing much energy and indirect pressure has succeeded in drawing attention to these needs, might not further be expected were there more doctors in Parliament? To place a man in the stifling atmosphere of party politics, and to make him discuss under party Whips vital questions at unhealthy hours is a strain that few men can survive, except perhaps doctors.

### PREMIER'S COUSIN RESIGNS A MEDICAL POST.

Sir Robert M. Simon, who has been for twenty-three years honorary physician to the General Hospital, Birmingham, has resigned his post. Sir Robert, who is a cousin of Mr. Asquith, is to have his services for the hospital commemorated, and a committee has been appointed to consider the best way of doing so. Sir Robert, who qualified in 1879, became M.D. of Cambridge in 1888 and M.D. of Birmingham University in 1910. Educated at Cambridge, Guy's Hospital, and Berlin, he has held the post of Professor of Therapeutics in Birmingham University, and has published several accounts of diseases incident to work in brass-foundries.

### THE CONTROL OF THE WORKHOUSE MASTER.

Mr. Astor recently elicited by a series of questions addressed to the President of the Local Government Board some very interesting information as to the provision made by Boards of Guardians for the treatment of the sick. In his reply the President differentiated between the provision made in workhouses and in separate infirmaries. Arranged in tabular form, the information given as regards beds for the sick was as follows:—

| | Less than 100 beds | 100 to 200 beds | 200 to 300 beds | Over 300 beds |
|---|---|---|---|---|
| In Workhouses... | 330 | 36 | 16 | 18 |
| In Separate Infirmaries......... | 144 | 41 | 27 | 59 |

It must be remembered that there are only about forty-five infirmaries completely separated administratively from the workhouses. The return shows, therefore, that about seventy-five Poor-Law institutions, each containing over 200 beds for sick patients, are under the supreme administrative control of the workhouse master. In commenting upon the provisions of the Poor-Law Institutions Order, we drew attention to the evils arising from the subordination of the medical officer to a lay official who was in no way qualified for the supervision of sick wards, but until these figures were published we doubt if anyone fully realised the extent of the evil. Now that Mr. Astor has obtained these facts, it is to be hoped that he will pursue his investigations, and will ask for a detailed return showing the number of beds for the sick in those workhouses and infirmaries in which the medical officer is in a subordinate position to the workhouse master.

### THE MENTAL HOSPITAL CHAPEL.

The Halifax Board of Guardians, under their new Chairman, the Rev. J. M. Walton, have passed a motion approving of a resolution from the Keighley Union, to the effect that they view with grave concern the Home Secretary's requisition, which called upon the West Riding Asylums Board to provide suitable chapels for the services at the Menston and Storthes institutions involving an expenditure of over £20,000. The usual discussion then followed on the relation of environment to religion, those in favour of the Keighley Union's resolution insisting that it was not so much economy which they had in view, but rather the assertion of the principle that, in Mr. J. B. Carter's words, "It was just as religious to worship in a barn as it is in a cathedral." Another speaker, Mr. J. H. Woodhead, said in the same vein that those who advocated ornamental additions to public worship were ecclesiastically-minded. This attempt to draw the red herring across the track fortunately did not impose for a moment on the Chairman, who, now in his twenty-first year of Poor-Law work, had all the experience of the best institutional administrators behind him when he said, while deploring the expenditure, that "medical men with experience of mental hospitals know that the patients should have a separate place of worship."

### THE RELATION OF ENVIRONMENT TO WORSHIP.

What economically-minded Guardians fail to see is that while it is certainly just as religious to worship in a barn as in a cathedral set apart for this express purpose for nine-tenths of the population, this rule does not and cannot apply to the staff and patients of institutions. To these service-time is recreation-time in the best sense; and just as the best administrators have found it essential to place the nurses' sleeping and recreation rooms away from the atmosphere of their work, so have they also found it essential to remove the atmosphere of routine work from the place where the services are celebrated. That is why a separate chapel is essential in institutional life. Only a little while ago we recorded an infirmary superintendent deciding that holding services in a committee room must be given up for this reason. He was right, and Mr. J. M. Walton is to be congratulated on seeing why he was so, and on not flinching from supporting the right principle, even though it happens to be very expensive now. Of course, chapels ought to have been provided in the first instance at the mental hospitals referred to.

### A NEW COLONY FOR THE FEEBLE-MINDED.

The Staffordshire Combination of Boards of Guardians has decided to purchase the Lovatt Estate for the purpose of establishing a colony for feeble-minded and epileptic persons. This estate comprises 120 acres at Wolverhampton, and includes a substantial building which has cost some £12,000, including the site. It is interesting to notice that the establishment of colonies of this class, though increasing in various parts of the country, has been particularly favoured by the Midlands, in which the Monyhull Colony for Epileptics, near Birmingham, is a well-known example. The difficult problems raised by the Mental Deficiency Act will give no doubt a further impetus to the colony movement, and the heart-searchings which, as recorded in The Hospital at the time, afflicted many local authorities, are beginning to settle down, and on the passing of that measure will soon afford by their results sufficient evidence to form an estimate of the value of the policy adopted.

### A ROYAL BIRTHDAY CAKE FOR CRIPPLED CHILDREN.

We may venture to think that there was unwonted joy among the crippled girls of the St. Agnes Home at East Croydon, who were visited by the Queen last month, when they received Princess Mary's birthday cake, which has been sent to the patients by the Queen. This is again only one of the kind acts which show the real sympathy and practical interest our Royal Family takes in institutional life and work.

### PRISON REFINEMENTS—OLD AND NEW.

When people used to die in prison, as often as not as the papers in the Record Office show, from starvation and neglect, it was always put down, said an authority recently, to natural causes. It was easy to draw a contrast and point out the relative cleanliness of present-day conditions, and still easier to snug oneself in a cloak of "progress"—a word of hypnotic effect on the modern lay mind—and to stop there. Granting that deaths from natural causes is a phrase used less loosely in this respect than it was once, let us test the "progress" of our prison system in another particular. Is torture still practised and still legal there? We should be glad to have our readers' views on the following case which recently came to our personal notice, and provides the sort of test case required. A certain man was sentenced to three months' imprisonment, with hard labour, eight years ago. He told us that he spent the first nights of his term on a plank bed in the ordinary way; and that the pain produced by it was intense. He added that, as the policy is gradually to decrease the rigour of the punishment, a few weeks later the plank bed gave way to a mattress, but only on alternate nights. This, he said, was a refinement of pain on that already endured. He did not know whether this system still obtained unaltered, but the point is: Does this plank bed, especially when used alternately with a mattress, amount to that degree of deliberately inflicted pain for punishment purposes which we call torture in our dictionaries? What is the view of the prison doctor and the private practitioner on this?

### SALVARSAN AND LAMBETH INFIRMARY.

It is interesting to note in the annual report of the medical officer of Lambeth Infirmary that there were 306 operations, as compared with 260 in the previous year. The number of insured patients treated was 506. Increased use was made of the salvarsan treatment. Altogether eighty injections were given. A patient suffering from cancer—aged thirty-eight—was treated, through the courtesy of the Radium Institute, with emanations of radium, which were supplied free of charge, and results were apparently most satisfactory. We understand that this was the only case of malignant disease admitted to the infirmary in which it was considered likely that radium would prove beneficial.

### NEW MEDICAL SUPERINTENDENT OF DOWNS SANATORIUM.

Dr. A. F. Cameron, M.A., M.D., C.M. (Edin.), D.P.H. (Camb.), has been promoted by the Metropolitan Asylums Board to the post of medical superintendent at the Downs Sanatorium. Dr. Cameron has served for several years as assistant medical officer at the South-Western and Southern Hospitals, and later as senior assistant medical officer at the South-Eastern Hospital. He has also acted as medical superintendent at the Southern and Joyce Green Hospitals for several months in each case. In December 1912, when the Board decided to use the Downs School as a sanatorium, they selected Dr. Cameron to act as medical superintendent at a salary of £450 per annum, with the usual allowances for board, lodging, etc. In view of the uncertainty of tenure it was not deemed advisable at that time to make any mention of an annual increase, but, having regard to recent events, the Board have decided to grant him the salary and emoluments of a superintendent, which are valued at £540 a year.

### A HOSPITAL'S EQUIPMENT.

The attention of every reader who takes a practical interest in the equipment of our hospitals and kindred institutions should be excited by the illustrated article on pages 161 to 164, entitled "The Practical Equipment of a Hospital." This is but the beginning of a new departure which we hope, with the aid of the most practical and experienced of our readers, will prove of real interest and permanent value as the scheme develops. It possesses the novelty of offering remunerative occupation to all practical workers with a little leisure who take a genuine interest in at least some of the sections into which the work of their hospital is divided. Much may be learnt and something may be gained by describing and illustrating some equipment of which they are most proud. Direct benefit may be derived by criticising the fittings or equipment which we may publish, and competing for a prize by filling in the voting-paper printed in another column. One of the most active and influential of our present hospital chairmen said this week, "No one can do without The Hospital." This new section and the competitions and practical workings it will include should demonstrate forcibly the truth of the fact expressed by the chairman in question.

### THE LEGAL RELATION OF GUARDIAN AND MEDICAL OFFICER.

Poor-Law medical officers sometimes have cause of complaint owing to individual members of Boards of Guardians assuming powers to which they have no right. The following short summary of the law on the subject may therefore be of interest to them. The word "workhouse" includes a separate Poor-Law infirmary. No Guardian has power to act in virtue of such office except as a member and at a meeting of the Board of Guardians (4 and 5 William IV., chap. 76, sec. 38). Any Guardian may, at any time, visit and examine any part of any workhouse of the Union or separate parish of which he is a Guardian (General Order, January 26, 1893). The new Poor-Law Institutions Order limits this power in the case of institutions to which the Order is applicable by making it subject to any general regulations made by the Guardians and by replacing the words "at any time" by "at reasonable hours." The Local Government Board have stated that they are not aware of any authority enabling an individual Guardian to introduce a visitor to the workhouse without the assent of the Board of Guardians. The Board has also stated that, although the Order of 1893 does not confer on a Guardian any express power to speak to any inmate when visiting the workhouse, the Board think he may do so if he desires, but that he should not in any way interfere with the discipline of the house or the authority of the head of the institution.

### IS WARD VENTILATION A MEDICAL MATTER?

The question of the right of an individual Guardian to examine books and documents has frequently been referred to the Local Government Board for decision, and that Board has always ruled that an individual Guardian has no such right except at meetings of the Boards of Guardians unless he has received specific directions authorising him to do so from the Guardians. The medical treatment and dieting of sick patients, including the use of stimulants; the ventilation and heating of the sick wards and similar matters are entirely under the control of the medical officer, and the Guardians, whether acting collectively or individually, have no power to interfere with them. Individual Guardians also have no power to demand information concerning the nature of the illness from which an inmate is suffering, and medical officers should exercise the greatest circumspection in answering such questions.

### A MEASURE OF SANATORIUM BENEFIT.

During the past six months 52,065 applications for sanatorium benefit under the Insurance Act were received in the United Kingdom. Treatment was accorded to 44,195 of these: the remainder are accounted for by 3,159, who were found not to be entitled to benefits on technical grounds connected with their contributions under the Acts, and by 4,711, who did not receive treatment on other grounds, such as erroneous diagnosis, refusal of the benefit offered, and those cases still under consideration when the six months expired. Of the 44,195 who were treated, 26,302 were received into institutions, 10,243 were treated at dispensaries, and 22,211 had domiciliary treatment: it is evident that about 14,600 persons had more than one form of treatment. Of the 26,302 sanatorium cases, 14,431 were discharged improved, 2,898 were discharged without improvement, and 1,638 died. The remainder are labelled "treatment discontinued" and "still under treatment." These figures reflect results which may be described as moderate: the large class of the improved would be a distinctly creditable achievement if it were certain that the meaning of the phrase is that the disease had been completely arrested and the patient rendered fit to resume his avocation. We doubt whether this interpretation would fit the facts of the case however. Excluding the cost of administration, the expenditure on sanatorium benefits was £651,600 in England, £103,200 in Scotland, £31,700 in Ireland, and £50,000 in Wales. It is evident that in Ireland there is either less tuberculosis among the insured, or less provision made for its relief and treatment. Probably the former is the correct explanation of the figures, in which case the inhabitants are not likely to regard their immunity as an " injustice."

### THE NEW PRINCIPAL OF LIVINGSTONE COLLEGE.

Dr. Loftus E. Wigram has been appointed by the committee of Livingstone College to succeed Dr. Charles F. Harford as principal, whose resignation takes effect at the end of July. Dr. Wigram, who was educated at Harrow, Trinity College, Cambridge, and St. Thomas's Hospital, is a graduate in medicine of Cambridge. Under the Church Missionary Society he was formerly a medical missionary at Peshawar on the North-West Frontier of India, and has thus had practical experience of missionary life abroad. He has been also for five years on the staff of Livingstone College, first as resident tutor and then as vice-principal, which comprises the chief feature for his new post of his experience in this country. Mrs. Wigram will be responsible for the household management hitherto carried out by Mrs. Harford. Thus they will maintain the tradition of the first twenty-one years of the life of the college, during which Dr. and Mrs. Harford have been in charge. Their staff of lecturers, includes Dr. Basil Price, who was for many years vice-principal of the college, and Mr. Alwyne Compton.

### LONDON MIDWIVES' INSPECTOR HONOURED.

The Midwives Act Committee of the London County Council have recently had under consideration a request from the Highlands and Islands Medical Service Board that Dr. Mary A. Pilliet, an inspector in the Public Health Department under the Midwives Act, may be permitted to undertake, in conjunction with Dr. Norman Walker, the Scottish representative on the General Medical Council, and Dr. Haultain, of Edinburgh, an inspection of the Cottage Nurses' Training Home in Govan, in connection with a proposal for the placing of subsidies from the Highlands and Islands Medical Service Fund at the disposal of certain nursing associations in the area under the Board's jurisdiction. The committee propose to grant Dr. Pilliet the necessary leave of absence for about a fortnight with full pay.

### PAYING PATIENTS AT TUBERCULOSIS DISPENSARIES.

The proposal to demand fees for treatment at the municipal tuberculosis dispensaries from persons whose incomes are £160 or more per annum is not meeting with general approval. The suggestion emanates from the London County Council, which recently expressed the view that such persons should be made to pay for treatment. The Council itself has no power to order payments, and has sent its suggestion to the Local Government Board, and also to the dispensary authorities of London. The Lambeth Borough Council has already concluded that it would not be in the best interest of the dispensaries to attempt to recover the costs from patients. In connection with the London County Council suggestion, Lambeth is deferring an expression of opinion pending the decision on the subject by the Local Government Board. The Public Health Committee, nevertheless, reports that the principle involved in the London County Council proposal is contrary to the spirit of the Lambeth dispensary scheme as approved by the Local Government Board and the Insurance Commissioners.

### THROAT HOSPITALS AMALGAMATION.

We are glad to note that the continuous, patient, long-suffering, helpful efforts of the Distribution Committee of the King's Fund have secured at last the amalgamation of the Hospital for Diseases of the Throat, Golden Square, with the London Throat Hospital, Great Portland Street. It is to be regretted that more of the throat hospitals have not entered into this scheme of amalgamation. We hope before the end of 1914 that the remaining throat hospitals will unite their forces and so secure that London may have the advantage of being served, so far as this speciality is concerned, by two well-found, thoroughly up-to-date and entirely efficient throat hospitals. It would be a real gain to secure the permanent exclusion and removal of relatively smaller and even inefficient so-called hospitals, which in the past have been a source of weakness rather than of strength to the medical institutions of London.

### COAL SAVING AT A MENTAL HOSPITAL.

In 1909 the London County Council authorised expenditure of £3,600 for the centralisation of the hot-water system at Cane Hill Mental Hospital, and the work was completed in June 1911 at a cost of £3,558. The new system has now been in operation for nearly three years, and the saving in the amount of steam coal used is shown by the following figures: Year ended March 1909, 3,584 tons; year ended March 1910, 3,608 tons; ten months ended March 1911, 2,908 tons—a total of 10,100 tons; ten months ended March 1912, 2,448 tons; year ended March 1913, 2,682 tons; year ended March 1914, 2,658 tons—a total of 7,788 tons. During the corresponding period, therefore, there has been an actual reduction in consumption of 2,312 tons, and a saving of £2,785. During the current year, it is expected that there will be a saving equal to £1,150, in which case the entire cost of the installation will have been more than met in the short period of less than four years.

### THE RELATION OF VENTILATION TO ORATORY

The inquiry, undertaken by Mr. O. W. Griffith, of the London Hospital, into the possibility of improving the ventilation of the House of Commons, has elicited, among others, the fact that its lack of freshness is due to the monotony of conditions existing in the Chamber. Mr. Griffith finds that, while the air is bacteriologically pure, it is not fresh, and he therefore suggests that a remedy might be found by changing the speed of the ventilating fans, and thereby altering the temperature at intervals. It is not fanciful to ask what relation ventilation and oratory bear to one another, nor whether Mr. Charles Stewart Parnell was not, unconsciously no doubt, hinting at the correspondence between the two when he asserted, in effect, that no independent party could be preserved in the House for a longer period than ten years, owing to the influence of its atmosphere. The moral of these investigations for the institutional world is, however, rather that they provide another instance of the relative failure of artificial systems of ventilation, which various well-known firms have tried to force upon hospitals of recent years. For instance, what has been the experience of the Royal Victoria Hospital, Belfast, where the Plenum system was installed some years ago? The question of pure air is like the question of pure milk—if it be artificially "sterilised," not merely the active harmful bacteria, but the organisms typical of "purity" are shown to run the risk of sharing a common fate.

### THE MEDICAL EXAMINATION OF WORKHOUSE ADMISSIONS.

The new Poor-Law Institutions Order is sometimes unjustly blamed by medical officers who have not carefully read its provisions. An instance occurred recently, in which the medical officer of a country workhouse wrote to his Board, saying that he objected to the new regulation by which he was required to see every case within an hour of its admission, even if there were no question of medical aid. He flatly refused to turn aside from a promised journey to a serious case just to race to the workhouse to a person who had not even complained of the least illness. If the medical officer in question had read the Order he would have been saved the necessity of writing the letter. The Order nowhere says an inmate shall be seen within an hour of admission. What the Order does say is that an inmate shall be examined as soon as practicable after admission by the medical officer. In this respect the new Order is indeed somewhat more considerate than the old Order replaced by it, which said it is the duty of the medical officer to examine the state of the paupers on their admission to the workhouse. This might reasonably be construed as requiring the immediate attendance of the medical officer. Medical officers have enough real grievances under the new Order without inventing imaginary ones.

### PRESS REPORTERS IN A HOSPITAL KITCHEN.

Princess Henry of Battenberg, who has consented to open the Pound Day at the Belgrave Hospital for Children in the Clapham Road, Kennington, has always evinced interest in the hospital since the day when she opened it some ten years ago. Her visit will be keenly enjoyed by those associated with it, more especially by those who were present when she opened the existing building. An amusing incident occurred on that occasion, which was not at the time recorded in the press. A party of reporters, the story goes, had sought refuge in the large kitchen whilst the Princess was being shown over the wards, and were making the best of the room. There were no chairs, and they had to find seating accommodation on the table, the only piece of furniture in the room. They were under the erroneous impression that the kitchen would not claim the attention of the Royal visitor, and were having a comfortable time when, much to their surprise, the Princess walked in. Enjoying their temporary consternation, she remarked, "Pray, don't disturb yourselves, gentlemen," a request which was not ignored, for the reporters, it is said, remained seated on the kitchen table whilst the Princess inspected the kitchen.

### THE BLIND AS ACTORS, AUTHORS, AND MUSICIANS.

The blind have been much before the public of late, owing to the interest shown in their welfare by the King and Queen, and also to Mr. Arthur Pearson's activities in procuring further Braille books for their instruction and entertainment. This interest will have served as a very useful kite to attract preliminary attention, and, therefore, added public support to the forthcoming International Conference and Exhibition, which is to be held at Church House, Westminster, from June 18 to 24 inclusive. Princess Louise was to open the conference, and a visit will be paid to the Royal Normal College, Upper Norwood, when a play and a concert will be given to visitors, in which a blind author, blind actors, and blind musicians will co-operate.

### ARTHRITIS RESEARCH AT BUXTON.

The investigations of the Cambridge Research Hospital, of which an illustrated account appeared in The Hospital on its opening a few years ago, lately again came before the public, and have led the Devonshire Hospital, Buxton, to prepare the way for its coming appeal for £25,000 by a reminder to the public, in the words of its patron, the Duke of Devonshire, that "a majority of its 316 beds are always occupied by patients suffering from rheumatoid arthritis in its various forms." He adds that "no difficulty is found in prevailing upon patients to submit to research." The hospital, which was established fifty-six years ago, is confronted with questions of reconstruction, and the sympathy which is being roused in the obscure disease which it exists to treat should open the purse of the public to the demand for money to carry out several overdue alterations which have now been practically decided on.

### CHAIRMAN'S GIFT OF A NEW HOSPITAL.

Few institutional officials, whether chairmen or otherwise, are in a position to make a free gift of a new hospital to their fellow-citizens, but this stricture of heart and purse does not apply to Mr. W. Cutlack, who presided over a recent committee meeting of the Ely Joint Hospital Board. In outlining the local position of affairs, and stating that Messrs. Macalister and Skipper, of Cambridge, and Mr. Wearing, of Norwich, the firms of architects to whom he applied, had respectively estimated the cost of building at £170 and £130 per bed. In support of his views that a new isolation hospital was a necessity for the neighbourhood, Mr. Cutlack is reported to have offered "to provide an isolation hospital with twelve beds, with administrative block, laundry, mortuary, etc., at an approximate cost of £130 per bed." The offer was unanimously accepted, and praise is due to the donor for his public spirit in proposing, as Chairman of the Ely Urban Council and also of the Joint Hospital Committee, to "give the hospital a start in the interests of the working-classes, who had so few chances of isolating children."

### A SEA-VOYAGE AS A "CURE" FOR CANCER.

Another success has crowned the activity of the Medical Defence Union by the conviction of a person for having obtained money by the false pretension of being a duly-qualified medical man. The prisoner, who was brought up at the London Sessions last week, according to the evidence, explained to a Roman Catholic priest that he was a doctor of medicine whose name did not appear on the Medical Register because through ill-health he was unable to practise. He further alleged that he could be reinstated by the payment of certain fees, amounting to some £10, which he induced the priest to advance. The prisoner also gave proof of his powers as a diagnostician by prescribing for the priest, whom he attended, a long sea-voyage as the best treatment for the "neurasthenia with symptoms of cancer," from which he asserted that his patient was suffering. After Dr. Bateman, secretary of the Medical Defence Union, had proved that the prisoner's name had never been upon the English Register, and further evidence had gone to show that he had "practised" in the rôles of schoolmaster, tutor, and bankrupt, and "graduated" already in prison for an offence similar to that with which he was charged, Mr. Wallace sentenced him to imprisonment with hard labour. The Medical Defence Union has been remarkably successful in the several cases which it has taken up, and its best claim to the support of the medical profession is the indication which these successes give of its good legal advice and wise administration.

### THE MARRIAGE OF FAITH AND SCIENCE.

In 1911 the special committee appointed at a conference of representatives of the clerical and medical professions to report upon the best methods of securing closer co-operation between the two professions adopted and published certain preliminary conclusions, and an enlarged committee was also formed. Its scope was defined under three headings, which were: To continue investigations into the meaning of "spiritual faith" and "mental" healing; to consider how the dangers connected with such treatment by persons not medically qualified might be guarded against; and to promote all legitimate co-operation between the two professions. This committee's report is now published by Macmillan at the popular price of one shilling (an indication at least of wisdom and good sense in those responsible for its drafting and publication), and we shall hope to review it in full at an early date. The Dean of Westminster was chairman of the committee, which included many distinguished names, Sir Douglas Powell, Sir Clifford Allbutt (honorary member), and the Dean of St. Paul's among them. In this Note we have room only for one quotation. It is this that, say the committee, "the physical results of what is called 'Faith' or 'Spiritual' healing do not prove on investigation to be different from those of mental healing or healing by 'suggestion.'" On page 235 of our issue of November 19, 1910, in a review of a volume on the same subject, we wrote: "Where, if anywhere, the dividing line can be drawn between mind and spirit, between suggestion and spiritual healing, is extremely obscure"; and, again: "Till the Church can draw some satisfactory distinction between the spirit and the mind we must look to the development of psychology to show us what the laws which underlie suggestion really are. Psycho-therapy must yield its secrets to scientific study, and the day when it has done so will be known by its inclusion as a formulated branch of treatment which can be applied as readily and usefully in certain cases by *anyone* who has qualified in his subject as, say, the surgical treatment of cataract." The report bears out our contention of five years ago in a striking manner.

### THIS WEEK'S DRUG MARKET.

A slight improvement in business has occurred. Cod-liver oil has an upward tendency in price, and a fair amount of business has been done; any substantial advance in price is hardly expected, but it might not be unwise to place orders at the rates now quoted. Refined camphor shows an upward tendency in price, and a definite advance in quotations would not come as a surprise. Cloves are unchanged, but a rise in value is not unexpected. The downward tendency in the price of cocaine is still noticeable. Eucalyptus oil is in very slow demand, but there is no quotable change in price at present. Essence of lemon shows no signs of improvement in price, and the general belief seems to be that the downward tendency may continue. The high price of American peppermint oil is well maintained. There appears to be no prospect of a decline in the value of olive oil, a movement in the other direction being more probable. There is little change in the position of opium. Jalap has a dearer tendency, as was anticipated; Mexican sarsaparilla also tends upwards in price. Unusually large supplies of drugs were offered at the recent public sales in Mincing Lane; the main features were a material decline in the price of cardamoms, a further drop in the value of menthol and Japanese peppermint oil, rather lower prices for honey and higher for nux vomica.

